# Analysis of microbial diversity in apple vinegar fermentation process through 16s rDNA sequencing

**DOI:** 10.1002/fsn3.944

**Published:** 2019-02-27

**Authors:** Juan Song, Ji‐Hong Zhang, San‐Jiang Kang, Hai‐Yan Zhang, Jing Yuan, Chao‐Zhen Zeng, Fang Zhang, Yu‐Long Huang

**Affiliations:** ^1^ Agricultural Product Storage and Processing Research Institute Gansu Academy of Agricultural Sciences Lanzhou China

**Keywords:** 16S rDNA, apple vinegar, fermentation, microbial diversity, physiological and biochemical characteristics

## Abstract

Based on SPME‐GC‐MS analysis, it could be found that the production of acetic acid, phenethyl acetate, and isoamyl acetate gradually increased in the apple vinegar fermentation broth with the fermentation time. Consequently, in order to systematically explore the dynamic changes of microbial diversity and metabolites in the process of apple vinegar fermentation, 16S rDNA were sequenced and analyzed in this work. The present results showed that bacterial diversity was rich and exhibited a certain variation during the dynamic fermentation process of apple vinegar. Furthermore, *Lactococcus* and *Oenococcus* were the predominant bacteria in the pre‐fermentation (alcoholic fermentation) of apple vinegar, while the dominant bacteria in the middle and late fermentation stages (acetic acid fermentation) were *Lactococcus* and *Acetobacter*. In addition, during the whole fermentation process of apple vinegar, *Lactococcus* was the most dominant bacteria, *Oenococcus* was the unique species in the stage of alcohol fermentation, and *Acetobacter* increased rapidly in the stage of acetic acid fermentation. In conclusion, our finding provided a theoretical basis for the processing technology of apple vinegar fermentation, and a theory evidence for the safety and health assessment of apple vinegar.

## 1 INTRODUCTION

Currently, healthy and safe fruit vinegar beverage is more and more favored by consumers with the improvement of people's living standards. Furthermore, apple vinegar was produced with apple juice through alcohol fermentation and acetic acid fermentation. It is a low‐cost and good flavor acid seasoning fruit vinegar beverage with high nutritional values. Additionally, previous works reported that apple vinegar has various potential pharmacological functions, such as antifungal properties, oral inflammation improvement (Mota, de Castro, de Araújo Oliveira, & de Oliveira Lima, 2015), hyperlipidemia prevention (Budak et al., 2011), disaccharide activity inhibition, and diabetes reduction. Studies have also shown that many functions of fruit vinegar drinks closely related to the microbial diversity in the fermentation process.

The 16S rDNA, a gene encoding 16S rRNA (a subunit of ribosomal RNA), includes conserved regions and hypervariable regions. The conserved regions are not significantly different among microbial species, while the hypervariable regions showed species specificity which altered with the difference of affiliation. At present, 16S rDNA sequencing technology has been widely used in microbial diversity research on animal flora, such as nematodes (Razia, Karthikraja, Padmanaban, Chellapandi, & Sivaramakrishnan, 2011), pigs (Qin et al., 2016), planthoppers (Yeh, Yang, & Hui, 2005), shrimps (Li, Xu, & Kou, 2014), fish (Pandey & Rajagopal, 2016), cattle and sheep (Gamal, Ahmed, Ahmed, & Teleb, 2016), bacterial and fungal pathogens, such as Cucumber wilt (Du et al., 2017), bacteremia (Alfonso et al., 2013), otitis media (Priit & Jelena, 2012), as well as soil microbial (Rana, Nidhal, & Abed, 2014), acid mine (Xie et al., 2011), and biological desulfurization (Lv et al., 2016). Besides, 16S rDNA is also considered as an important indicator for the classification of microorganisms in fermented foods such as industrial vinegar (Trček, Lipoglavšek, & Avguštin, 2016; Trček, Mahnič, & Rupnik, 2016), Chinese cereal vinegar (Li et al., 2016), grain vinegar (Wang, Zhang, & Gui, 2015), coconut vinegar (Mohamad, Yeap, & Ky, 2017), wine‐soaked vinegar (Trček et al., 2016, 2016), apple vinegar (Štornik, Skok, & Trček, 2016), and other food microbial fermentation products. However, there are few researches about 16S rDNA analysis performed on the microbial diversity during apple vinegar fermentation.

In the present study, we constructed a high‐throughput sequencing library by 16S rDNA to identify the diversity of microbial flora during the dynamic fermentation of apple vinegar. Our results provided a theoretical basis for the processing technology of apple vinegar fermentation, and a theory evidence for the safety and health assessment of apple vinegar.

## 2 MATERIALS AND METHODS

### 2.1 Materials and strains

Fuji apples used in this research collected from Qingyang, Gansu province, China. Yeast strains *Saccharomyces cerevisiae* CICC1750 and acetic acid bacteria *Acetobacter pasteurianus* CICC20056 were purchased from China microbial culture preservation Center (Beijing, China).

### 2.2 Media preparation

Potato medium: potato 200 g, glucose 20 g, tap water 1,000 ml, pH 6; Liquid medium: glucose 1%, yeast powder 1%, anhydrous alcohol 3%, pH 4.5; Solid medium: glucose 1%, yeast powder 1%, absolute alcohol 3%, agar 2%, pH 4.5.

### 2.3 Bacterial activation

The dissolved bacteria suspension was transferred to a tube containing 4–5 ml liquid medium, mix, and take 100 μl transferred to a solid medium, a generation of bacteria to be the extension of training time, transfer to 2–3 generations to restore vitality.

### 2.4 Apple juice processing

Fuji apples were crushed after washing, and then, the apple juice was prepared and subsequently prepared by 0.04% pectinase. Then, the apple juice was digested at 45°C for 1 hr and sterilized at 90°C for 1.0 min.

### 2.5 Apple vinegar producing technology

After sterilization, the apple juice was filtrates. For the alcohol fermentation stage, 8% activated yeast liquids were added into 1,000 ml apple juice. Then, the mixture samples were fermented statically at 24°C. Then, the alcohol fermentation was stopped until the alcohol concentration was no longer changed (the alcohol content was determined as described in the following part of 2.5), and the acetic acid fermentation was started. The 10% activated acetic acid bacteria solution was added and shaking cultured with 120 rpm/min at 28°C, and the acetic acid fermentation was stopped until the concentration of acetic acid kept stable (the acetic acid content was determined as described in the following part of 2.5).

After 8 days of alcohol fermentation and 8 days of acetic acid concentration, the fermented fluids were filtrated and sterilized. Subsequently, the sterilized fermented fluids were placed statically in airtight container for approximately 7 days to afford the apple vinegar.

### 2.6 Alcohol and acetic acid determination using SPME‐GC‐MS assay

Contents of alcohol and acetic acid were determined by using headspace solid phase micro‐extraction gas chromatography–mass spectrometry (SPME‐GC‐MS) assays according to the method reported by Wang, Wang, Liu, Liu, and Fan, (2012). Briefly, volatile substances including alcohol and acetic acid were extracted by a DVB/CAR/PDMS probe of the TriPlus‐RSH‐Autosampler‐SPME system. A DB‐WAX capillary column (30 m × 0.25 mm × 0.25 μm) was used for the sample separation, and the temperature program carried out was as follows: the initial temperature was 40°C for 2 min, which was increased to 180°C at 5°C/min; then, the temperature increased to 230°C at 15°C/min. Helium (99.999%) was used as carrier gas kept at a flow rate of 1.2 ml/min. The split rate was set as 40:1, and inlet volume was set as 1.0 μl. Electron impact ionization (EI) was used, and the ion energy was set as 70 eV with the mass range scanned was 33.00–350.00 u in full‐scan acquisition mode. Alcohol and acetic acid were identified by comparing the mass spectra with the standard references and quantified using internal standard method.

### 2.7 MetaVx™ library construction and Illumina MiSeq sequencing

High‐throughput sequencing library construction and sequencing based on Illumina MiSeq platform was carried out by GENEWIZ Corporation (Suzhou, China). DNA samples were tested for concentration using the Qubit 2.0 Fluorometer, and a sequencing library was constructed using the MetaVx™ library construction kit. Using 30–50 ng of DNA as a template, the PCR primers used were “CCTACGGRRBGCASCKVRVGAAT” sequence and a downstream primer comprising the “GGACTACNVGGGTWTCTAATCC” sequence, and two highly variable regions including V3 and V4 on the 16S rDNA of the prokaryote were amplified, and the end of the PCR product of 16S rDNA Index linker for NGS sequencing. Library quality was detected using an Agilent 2100 bio‐analyzer, and library concentrations were detected by a Qubit 2.0 Fluorometer. After hybridization of DNA libraries, 2 × 300 bp double‐ended sequencing (PE) was performed by Illumina MiSeq and the sequence information was read by MiSeq's own MiSeq Control Software (MCS).

### 2.8 Biological information analysis

Based on the OTU analytical results, Shannon, Chao1, and other α‐diversity indices were analyzed for each sample separately to obtain the information of species richness and evenness of each sample. Based on taxonomic information, the statistical analysis of community structure was carried out at each classification level. Through the NMDS analysis, we constructed the clustering tree of UPGMA (Unweighted pair group method), which showed the difference of community structure between different samples or groups.

### 2.9 Statistical analysis

The experiments were repeated a minimum of three times. All data were expressed as means ± *SD*. Statistical differences between control and treated groups were evaluated using Student's *t* test, and differences between groups were considered statistically significant at *p* value <0.05.

## 3 RESULTS AND DISCUSSION

### 3.1 Results of biochemical and physiological factors assay during apple vinegar fermentation

**Figure 1:**
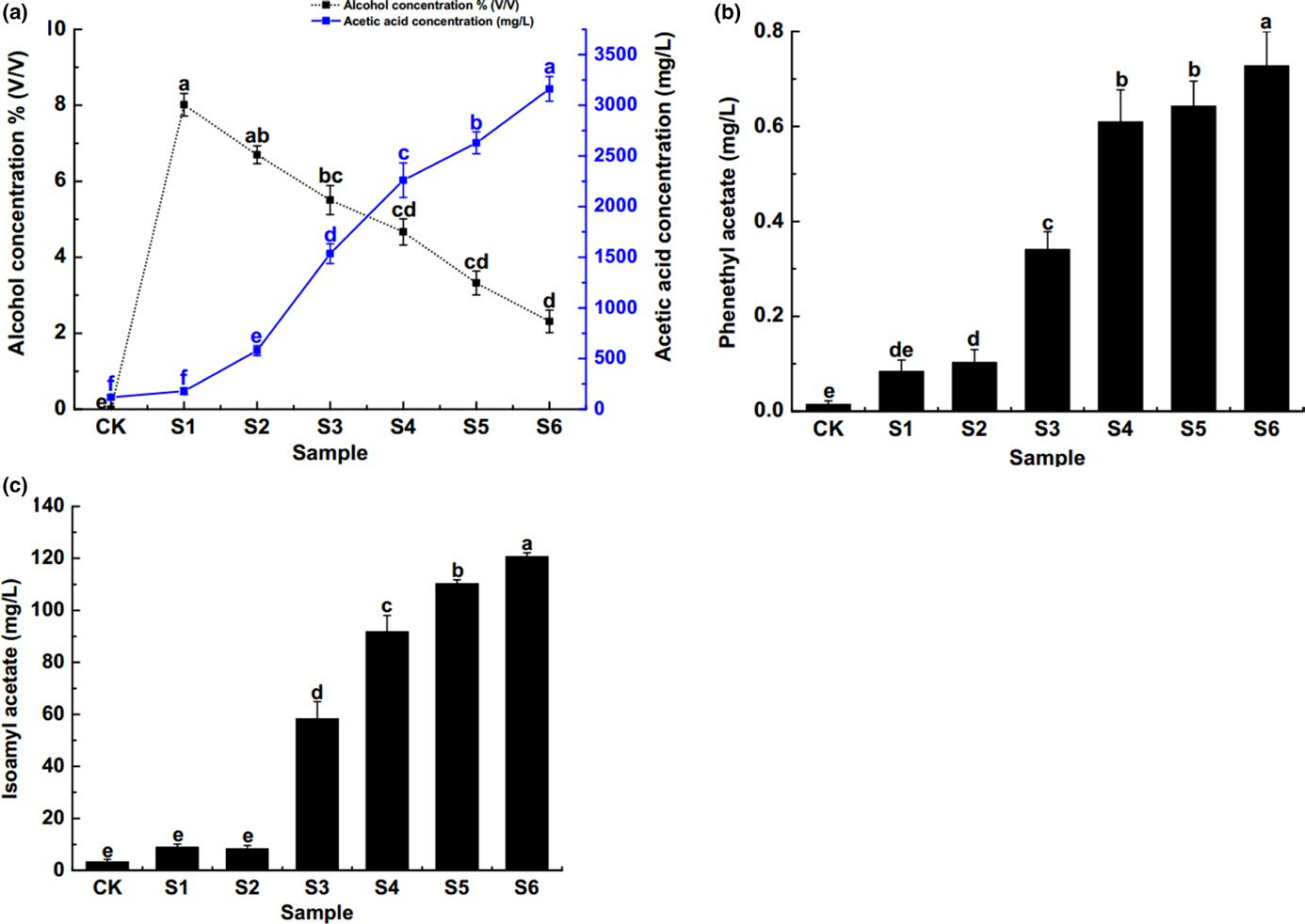
Physiological and biochemical detection during fermentation. (a) The relationship between alcohol concentration and concentration of acetic acid during the fermentation of apple vinegar. (b) The concentration of ethyl acetate during the fermentation of apple vinegar. (c) The concentration of isoamyl acetate during the fermentation of apple vinegar. Same a‐f indicates that there is no significant difference between different samples during the fermentation process (*P* > 0.05), while the difference is significant (*P* < 0.05)

Alcohol and acetic acid contents were determined in different periods during the fermentation of apple vinegar by CK (0d), S1 (3d), S2 (7d), S3 (9d), S4 (10d), S5 (12d), and S6 (14d). Results indicated that the highest alcohol content appeared at S1 and then declined sharply while the acetic acid concentration gradually increased and reached peak at S6, which showed that the alteration of alcohol concentration and acetic acid concentration showed a negative correlation during the fermentation of apple vinegar (Figure 1a). This phenomenon mainly caused by the interaction of various microorganisms in the process of apple vinegar fermentation, which regulated alcohol fermentation and acetic acid fermentation of the entire vinegar production phase by turning glucose into alcohol at first, and then oxidizing alcohol into acetic acid. We also found that phenylethyl acetate (Figure 1b) and iso‐amyl acetate (Figure 1c) production was significantly increased with the extension of fermentation time, indicating that S6 contains the most characteristic aroma components and flavors.

### 3.2 16S rDNA sequencing

Samples of CK, S1, S2, S3, S4, S5, and S6 in the fermentation process of apple vinegar were used to construct the 16S rDNA clone library for bioinformatics analysis. V3 and V4 regions of 16S rDNA gene during the fermentation of apple vinegar were sequenced and performed data amount and quality optimization statistics (Table 1). All sequences were clustered into 29 OTU, and all the OTU were classified as bacterial community by using Qiime (1.9.1) and Vsearch (1.9.6) analysis software (Table 2).

**Table 1:** Sequencing raw data quality statistics

Sample	Nucleic acid concentration	Reads	Nochimera	Sample	Nucleic acid concentration	Reads	Nochimera
CK_1	4.520	119476	53059	S4_1	0.458	223438	101334
CK_2	0.840	152638	67470	S4_2	0.366	107226	47216
CK_3	0.418	137928	60832	S4_3	0.380	96456	42367
S1_1	3.100	162776	73809	S5_1	0.458	153152	68445
S1_2	2.040	114578	50808	S5_2	0.624	108450	47603
S1_3	3.940	109658	48150	S5_3	0.386	110232	48772
S2_1	0.610	139038	60656	S6_1	0.120	125710	56283
S2_2	0.542	111092	48110	S6_2	12.800	198876	91673
S2_3	1.010	95896	41801	S6_3	11.100	155922	71576
S3_1	0.398	171454	75488				
S3_2	0.390	131512	58123				
S3_3	0.510	138950	61216				

**Table 2:** OTU table

ID	Taxonomy
OTU01	k__Bacteria; p__Firmicutes; c__Bacilli; o__Lactobacillales; f__Lactobacillaceae; g__Lactobacillus; s__Lactobacillus_farraginis
OTU02	k__Bacteria; p__Proteobacteria; c__Gammaproteobacteria; o__Enterobacteriales; f__Enterobacteriaceae
OTU03	k__Bacteria; p__Firmicutes; c__Bacilli; o__Lactobacillales; f__Lactobacillaceae; g__Lactobacillus; s__Lactobacillus_plantarum
OTU04	k__Bacteria; p__Firmicutes; c__Bacilli; o__Lactobacillales; f__Lactobacillaceae; g__Lactobacillus; Ambiguous_taxa
OTU05	k__Bacteria; p__Proteobacteria; c__Alphaproteobacteria; o__Rhodospirillales; f__Acetobacteraceae; g__Acetobacter; Ambiguous_taxa
OTU06	k__Bacteria; p__Firmicutes; c__Bacilli; o__Lactobacillales; f__Leuconostocaceae; g__Oenococcus; Ambiguous_taxa
OTU07	k__Bacteria; p__Proteobacteria; c__Gammaproteobacteria; o__Enterobacteriales; f__Enterobacteriaceae; g__Escherichia‐Shigella; Ambiguous_taxa
OTU08	k__Bacteria
OTU09	k__Bacteria; p__Firmicutes;c__Bacilli; o__Lactobacillales; f__Lactobacillaceae; g__Lactobacillus; s__Lactobacillus_casei
OTU10	k__Bacteria; p__Proteobacteria; c__Gammaproteobacteria; o__Enterobacteriales; f__Enterobacteriaceae
OTU11	k__Bacteria; p__Proteobacteria; c__Gammaproteobacteria; o__Enterobacteriales; f__Enterobacteriaceae
OTU12	k__Bacteria; p__Firmicutes;c__Bacilli; o__Lactobacillales; f__Lactobacillaceae; g__Lactobacillus; s__Lactobacillus_salivarius
OTU13	k__Bacteria; p__Proteobacteria; c__Betaproteobacteria; o__Burkholderiales; f__Comamonadaceae; g__Variovorax; Ambiguous_taxa
OTU14	k__Bacteria; p__Firmicutes; c__Bacilli; o__Bacillales; f__Bacillaceae; g__Bacillus
OTU15	k__Bacteria; p__Gemmatimonadetes; c__S0134_terrestrial_group; o__uncultured_Gemmatimonadetes_bacterium; f__uncultured_Gemmatimonadetes_bacterium; g__uncultured_Gemmatimonadetes_bacterium; s__uncultured_Gemmatimonadetes_bacterium
OTU16	k__Bacteria; p__Chlorobi; c__Chlorobia; o__Chlorobiales; f__OPB56
OTU17	k__Bacteria; p__Actinobacteria; c__Actinobacteria; o__Frankiales; f__Sporichthyaceae; g__hgcI_clade
OTU18	k__Bacteria; p__Proteobacteria; c__Betaproteobacteria; o__Burkholderiales; f__Burkholderiaceae; g__Limnobacter
OTU19	k__Bacteria; p__Proteobacteria; c__Betaproteobacteria; o__Methylophilales; f__Methylophilaceae; g__Candidatus_Methylopumilus; s__uncultured_bacterium
OTU20	k__Bacteria; p__Fusobacteria; c__Fusobacteriia;o__Fusobacteriales; f__Fusobacteriaceae; g__Cetobacterium
OTU21	k__Bacteria; p__Proteobacteria;c__Gammaproteobacteria;o__Pseudomonadales;f__Moraxellaceae;g__Acinetobacter
OTU22	k__Bacteria; p__Bacteroidetes; c__Flavobacteriia; o__Flavobacteriales; f__Flavobacteriaceae; g__Chryseobacterium; Ambiguous_taxa
OTU23	k__Bacteria; p__Actinobacteria; c__Thermoleophilia; o__Solirubrobacterales
OTU25	k__Bacteria; p__Bacteroidetes; c__Flavobacteriia; o__Flavobacteriales; f__Flavobacteriaceae; g__Myroides; s__uncultured_bacterium
OTU26	k__Bacteria; p__Verrucomicrobia; c__Opitutae; o__Opitutales; f__Opitutaceae; g__Opitutus; Ambiguous_taxa
OTU27	k__Bacteria; p__Proteobacteria; c__Alphaproteobacteria; o__Rickettsiales; f__Rickettsiales_Incertae_Sedis; g__Candidatus_Finniella;Ambiguous_taxa
OTU28	k__Bacteria; p__Bacteroidetes; c__Bacteroidetes_Incertae_Sedis; o__Order_III; f__BIgi5; g__uncultured_bacterium; s__uncultured_bacterium
OTU29	k__Bacteria; p__Bacteroidetes; c__Sphingobacteriia; o__Sphingobacteriales; f__NS11‐12_marine_group; g__uncultured_bacterium; s__uncultured_bacterium

### 3.3 Species annotation

**Figure 2:**
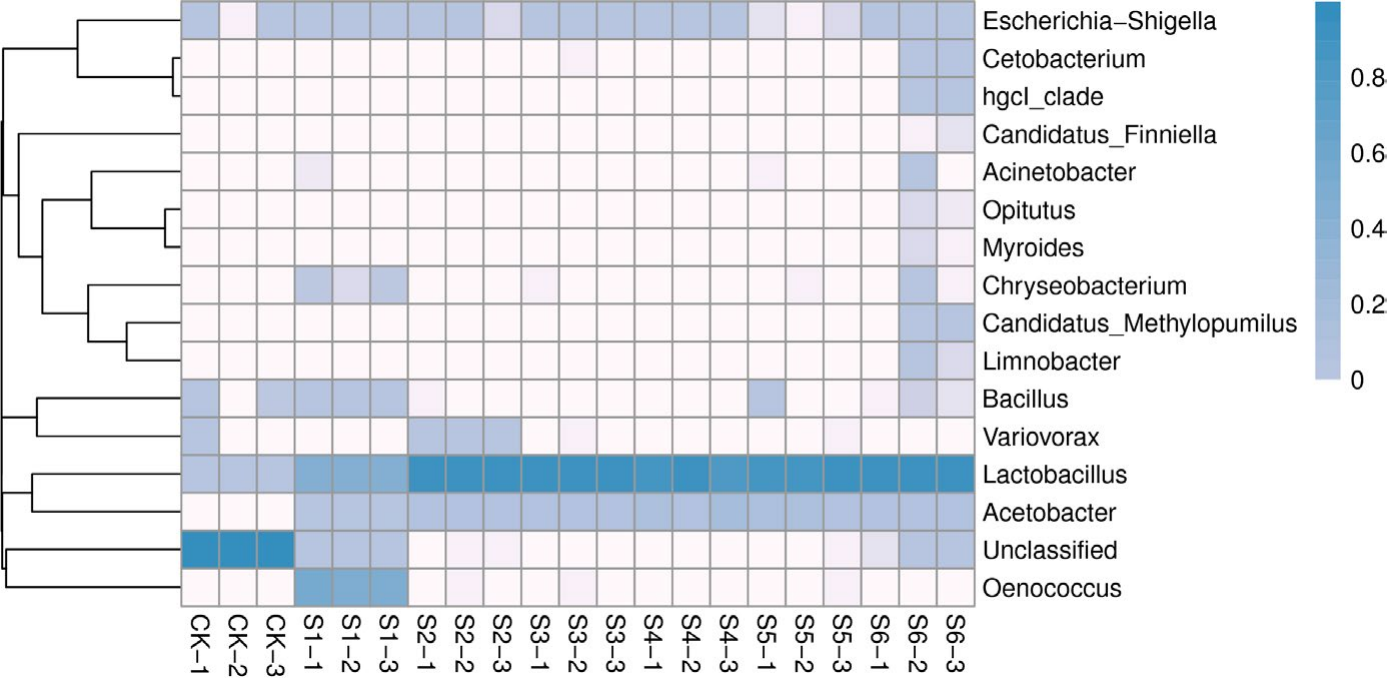
Heat map of species distribution. Column names as samples information, the name for the species name, the figure at the top of the tree as samples clustering, clustering tree species figure on the left, and the middle heat maps each grid corresponding to different color value for each row of the relative abundance of species

To obtain the taxonomic information of OTU, a representative sequence was selected for each OTU, and the representative sequence was annotated by species using RDP classifier. Then, the community composition of each sample was obtained by Qiime (1.9.1) software analysis. The result showed that the dominant bacteria in the dynamic fermentation of apple vinegar are *Lactococcus*,* Oenococcus* and *Acetobacter*. Among them, there are obvious differences in the composition of microbial community between the control group and the treatment group; however, there is also certain regularity (Figure 2). Compared to control CK, the main bacterial species in treatment groups S1, S2, S3, S4, S5, and S6 was *Lactococcus*, and its number tended gently with the increase in fermentation time, which showed that *Lactococcus* is the key microorganisms in the fermentation process of apple vinegar. However, *Oenococcus*, uniquely contained at S1, is also the dominant bacterium, indicating that the early fermentation of apple vinegar belongs to alcoholic fermentation.

There is *Acetobacter* with strong acid‐producing ability in S2, S3, S4, S5, and S6 samples, which quantity increased sharply and then decreased slightly with the prolongation of fermentation time. *Acetobacter* was multiplied in the middle of acetic acid fermentation, indicated that it was the secondary dominant bacterium in the fermentation of apple vinegar. Specially, the species in S6 was the most abundant; which indicated that there are many kinds of microorganisms in the late fermentation of acetic acid, which is of great significance to the unique taste and scent of apple vinegar.

### 3.4 Sample complexity analysis

In community ecology, α‐diversity is mainly concerned with the single sample diversity analysis, which can reflect the number of species in the microbial communities. The species richness and diversity of environmental communities can be estimated by the analysis of Chao1, Shannon, Simpson, and good's coverage indices (Table 3). Compared with the control group, the Chao1 index of the S6 sample of apple vinegar was significantly higher than that of the other samples, while the Simpson index was significantly lower than that of the other samples, indicating that the microbial community differed greatly in the later stage of apple vinegar fermentation. The Shannon index of sample S2 was significantly higher than that of other samples, indicating that there was a great difference in microbial community in sample S2. These results were consistent with the results of subordinate level classification statistics and species distribution heat map analysis. The good's coverage index of all the samples is 1, that is, the coverage of the sample reaches 100%, indicating that the coverage of the sample library is very large, and the probability that the sample has not been detected in this sequence is very low, which indicates that this sequencing library has very good representation.

**Table 3:** Collation of alpha diversity results

Sample	Chao 1	Shannon	Simpson	GC	Sample	Chao 1	Shannon	Simpson	GC
CK_1	8.0	0.033	0.006	1	S4_1	6.0	1.127	0.427	1
CK_2	3.0	0.006	0.001	1	S4_2	6.0	0.968	0.355	1
CK_3	5.0	0.009	0.001	1	S4_3	6.0	1.218	0.458	1
S1_1	14.0	1.212	0.520	1	S5_1	8.0	1.101	0.406	1
S1_2	13.0	1.233	0.527	1	S5_2	8.0	1.069	0.398	1
S1_3	14.0	1.219	0.524	1	S5_3	12.0	0.968	0.354	1
S2_1	8.0	1.332	0.526	1	S6_1	7.0	0.862	0.300	1
S2_2	10.0	1.160	0.461	1	S6_2	22.0	1.152	0.354	1
S2_3	8.0	1.359	0.558	1	S6_3	20.5	0.903	0.273	1
S3_1	7.0	0.994	0.378	1					
S3_2	15.0	0.865	0.314	1					
S3_3	6.0	0.959	0.362	1					

GC: good coverage.

### 3.5 Rarefaction curve

**Figure 3:**
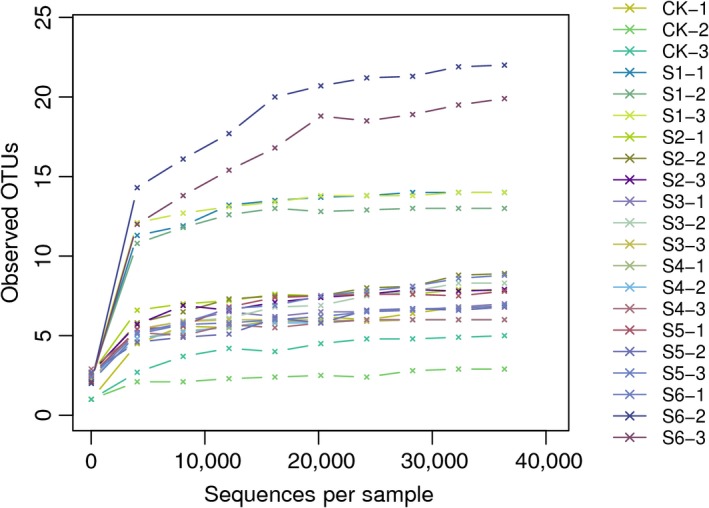
OTUs rarefaction curves. The x‐coordinate is the number of effective Sequences, and the ordinate is the number of OTU. Each curve in the graph represents a sample with different color marks; Sequence depth increase the number of OTU. When the curve flattens out, the number of OTU detected is no longer increased with the increase in the amount of data extracted, and the sequencing data are reasonable

The rarefaction curve is widely used to determine whether sample size is adequate to estimate species richness. The results showed that as the depth of sequencing increases, the number of OTUs increases and the rarefaction curve tends to be flat and eventually reached the plateau which sequencing data can reflect the dynamic process of apple vinegar bacterial diversity (Figure 3).

### 3.6 Significant analysis of differences in the structure of community groups

**Figure 4:**
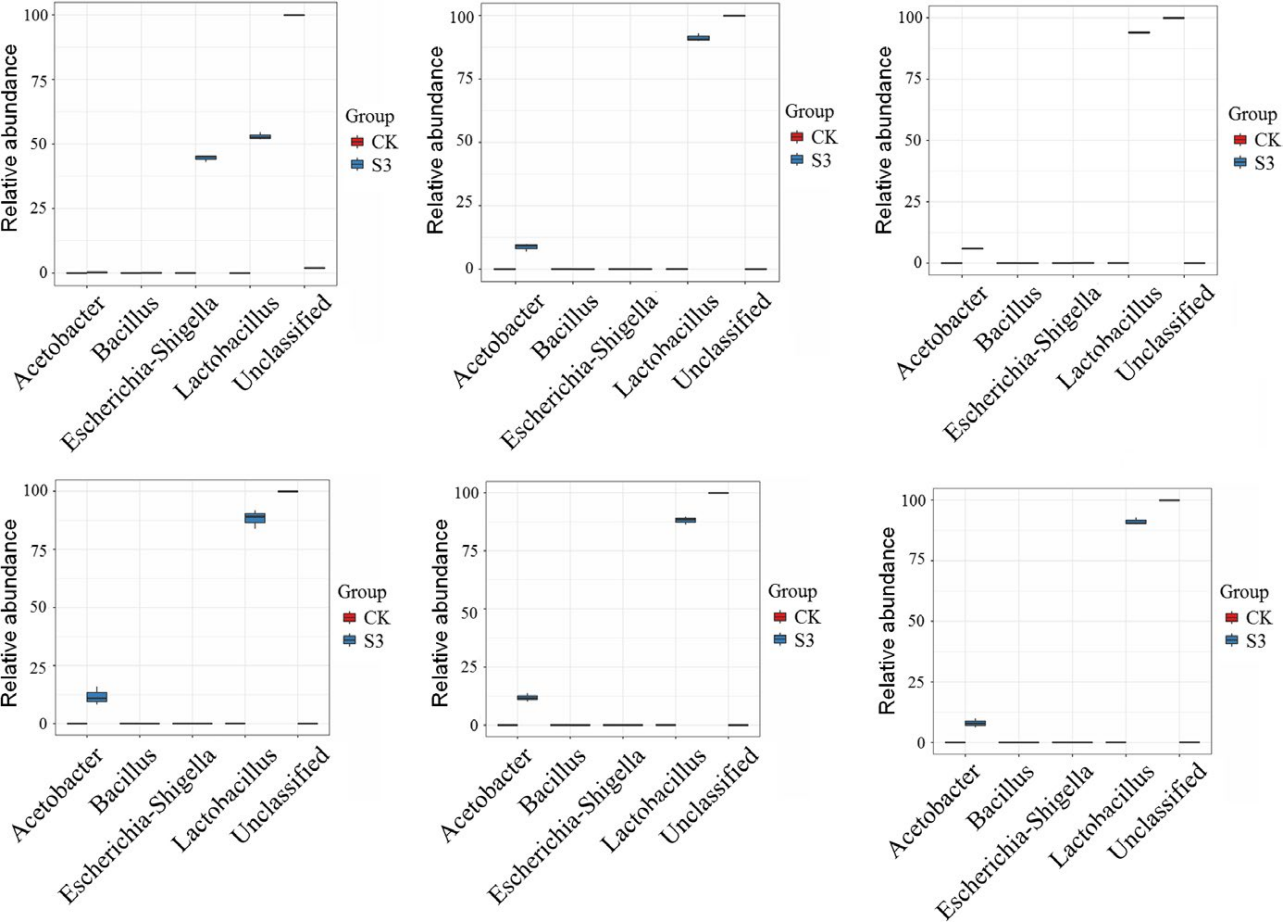
Differentially abundant features. In the figure, the abundance distribution of the five species with the largest difference in the two groups is shown, and the horizontal coordinates are the five species classification names with the largest difference between the two groups, and the vertical coordinates are the relative abundance of the species

Significant difference analysis of species composition among groups could be performed according to the community abundance data of different groups, and the strict statistical method can be used to detect the classification of abundance difference between the two microbial communities. The multiple hypothesis tests and false discovery of rare and the frequency data rate (FDR) analysis can assess the significance of the observed differences. From the Metastats difference map (Figure 4), we know that there are significant differences in the abundance of *Lactococcus*,* Oenococcus* and *Acetobacter* among the dominant species in all the samples compared with the control group. There are also insignificant differences in abundance, which is consistent with the results of species distribution heat map.

### 3.7 Multi‐sample comparative analysis

**Figure 5:**
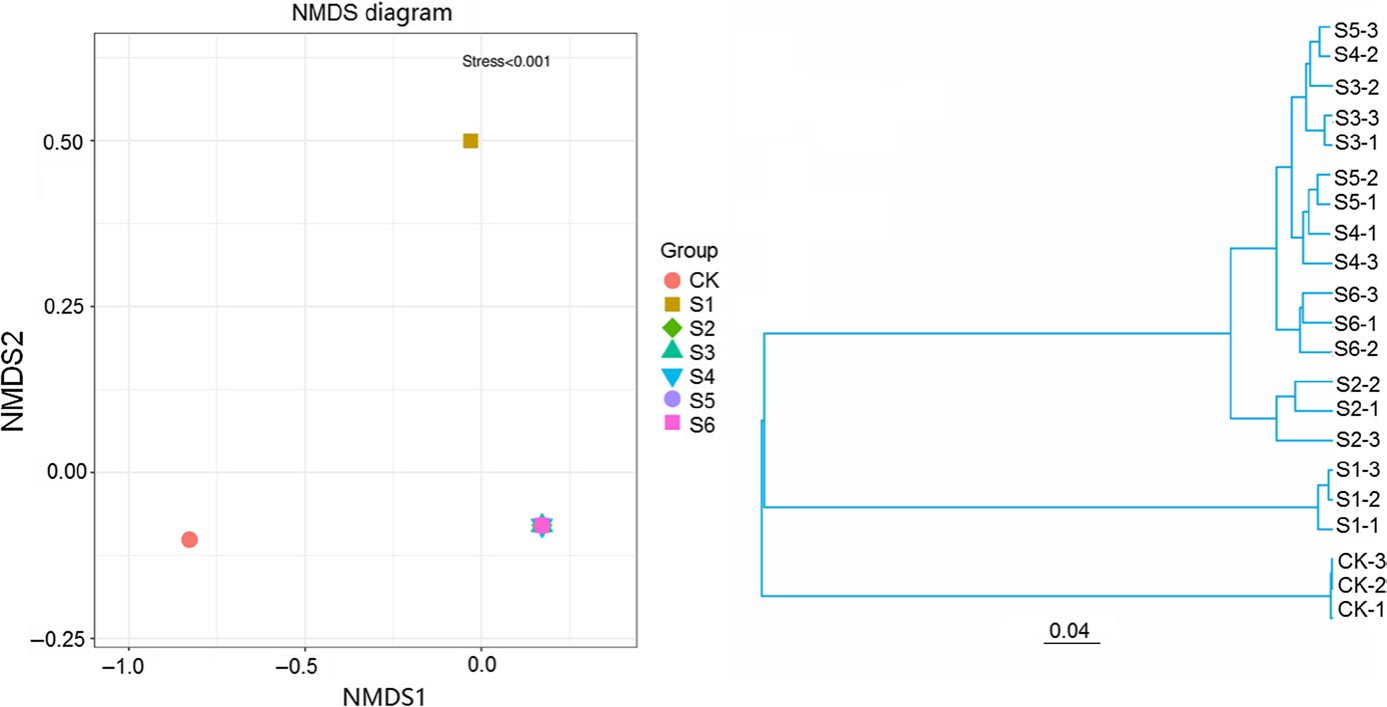
Multi‐sample comparative analysis. NMDS Plot. Each point in the graph represents a sample, the distance between the point and the point indicates the extent of the difference, and the sample of the same group uses the same color representation. When stress is less than 0.2, NMDS can accurately reflect the differences between samples. UPGMA tree. Each branch in the graph represents a sample, with different colored branches representing different groups

Non‐metric multidimensional scaling (NMDS) method is a data analysis method that simplifies the research object in multidimensional space to location, analysis and classification in low‐dimensional space while preserving the original relationship between objects. Results indicated that the difference became larger with decreasing of the similarity. S2, S3, S4, S5, and S6 samples are almost stacked together, indicating that the similarity between them is higher and the difference is smaller. It shows that NMDS can accurately reflect the degree of difference between samples when stress < 0.2 (Figure 5a).

Based on the Bray‐Curtis distance matrix, samples were clustered using UPGMA to analyze microbial community differences. We found that all samples were copolymerized into two categories (Figure 5b). The first category is the control CK group, and the second category includes alcohol fermentation process of S1 and S2 and acetic acid fermentation process S3, S4, S5, and S6. Among the second category, S1 is clustered alone, indicating that the microorganisms diversity in the pre‐alcoholic fermentation are relatively high compared with other fermentation stages, and S2 was also clustered together, indicating that microbial diversity in the late stage of alcoholic fermentation was larger than that in the acetic fermentation stage. S2 and S6 are clustered together, indicating that the microorganisms in the group have low similarity and large difference. However, the branches in S4 and S5 groups are slightly interspersed, indicating that there is a high similarity between the microbial communities in acetic acid fermentation medium with a small difference.

## 4 CONCLUSION

The present results of SPME‐GC‐MS showed that the production of acetic acid, phenethyl acetate, and isoamyl acetate in the apple vinegar fermentation broth gradually increased with the fermentation time of apple vinegar. Analysis of microbial diversity in apple vinegar fermentation process, which performed through 16S rDNA high‐throughput sequencing, showed that the bacterial diversity of apple vinegar during the dynamic fermentation process is rich and has some changes. *Lactococcus* and *Oenococcus* were the predominant bacteria in the early stage of apple vinegar fermentation (alcoholic fermentation). The number of *Lactococcus* tended to be gentle after the rapid increase with the prolongation of fermentation. The main dominant bacteria in the middle and late stages of fermentation (acetic acid fermentation) were *Lactococcus Acetobacter*. In addition, the number of *Lactococcus* and *Acetobacter* decreased slightly after a sharp increase with the prolongation of fermentation. These findings explained the potential reasons of the gradual increase in acetic acid, phenethyl acetate, and isoamyl acetate during the dynamic fermentation of apple vinegar. Besides, we think the main reason might be that a variety of microorganisms synergistically formed the unique taste and flavor of the fermented vinegar products. In conclusion, our present study systematically investigated the microbial diversity during the dynamic fermentation of apple vinegar and provided some theoretical basis for the health and safety evaluation of the vinegar and its products.

## CONFLICT OF INTEREST

The authors declare that they have no conflict of interest.

## ETHICAL STATEMENT

The study did not involve any human or animal testing.
